# Epigallocatechin gallate attenuates tumor necrosis factor (TNF)-α-induced inhibition of osteoblastic differentiation by up-regulating lncRNA TUG1 in osteoporosis

**DOI:** 10.1080/21655979.2022.2056825

**Published:** 2022-03-31

**Authors:** Yanfeng Han, Dening Pei, Wenjing Li, Bin Luo, Qingsong Jiang

**Affiliations:** a Department of Implant, School of Stomatology, Capital Medical University, Beijing, Hebei, China; b National Institutes for Food and Drug Control, Beijing, Hebei, China; c Department of Prosthodontic, School of Stomatology, Capital Medical University, Beijing, 100050, China

**Keywords:** Osteoporosis, osteoblasts, epigallocatechin gallate, lncRNA TUG1, TNF-α

## Abstract

Promoting osteoblast proliferation and differentiation contributes to the prevention and clinical treatment of osteoporosis. This study was to investigate the effect and mechanism of epigallocatechin gallate (EGCG) on tumor necrosis factor (TNF)-α-caused inhibition of osteoblastic differentiation. First, we cultured mouse embryo osteoblast precursor cells (MC3T3-E1) and induced by TNF-α (0, 2.5, 5, 10 ng/mL). The results revealed that TNF-α significantly inhibited the proliferation, ALP activity and mineralized nodule formation of MC3T3-E1 cells and promoted apoptosis. However, EGCG pretreatment significantly alleviated the inhibitory effect of TNF-α on MC3T3-E1. In addition, TNF-α significantly downregulated the expression of lncRNA TUG1 in MC3T3-E1, while EGCG upregulated the expression of lncRNA TUG1. After overexpression of lncRNA TUG1 in TNF-α-induced MC3T3-E1 cells, it could show similar effects as EGCG. However, interference with lncRNA TUG1 expression diminished the protective effect of EGCG on TNF-α-induced MC3T3-E1 cells. Finally, we found that EGCG inhibited TNF-α-induced activation of the Hippo/YAP signaling pathway, and that low expression of lncRNA TUG1 suppressed this effect. In conclusion, EGCG could suppress Hippo/YAP pathway activity by up-regulating lncRNA TUG1, ultimately improving TNF-α-caused inhibition of osteoblastic differentiation.

## GRAPHICAL ABSTRACT

**Figure:**
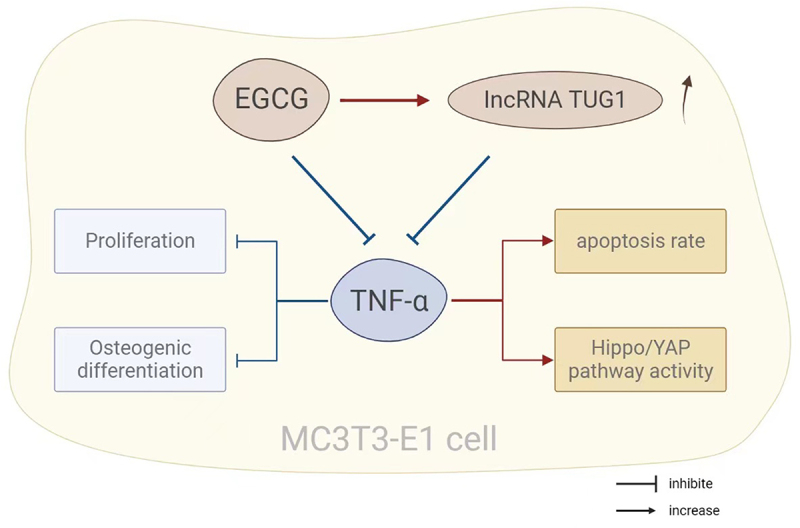

## 1. Introduction

As a bone disease, osteoporosis is characterized by skeletal fragility and increased risks of fractures due to an imbalance in bone homeostasis [1]. Two major forms of osteoporosis are primary osteoporosis and secondary osteoporosis. Primary osteoporosis occurs because of the cessation of menopausal women for estrogen production, while secondary osteoporosis, because of metabolic diseases and disorders, organ dysfunction, malnutrition, and lifestyle habits [2]. The latter is also a manifestation of the side effect of using drugs [2]. The development of osteoporosis is mainly attributed to the occurrence of a great quantity of hematopoietic and immune factors in the bone microenvironment. The hematopoietic and immune factors are complex and interacting, affecting bone formation and resorption [3]. Tumor necrosis factor-α (TNF-α), not only the strongest bone resorption promoter but also bone formation inhibitor, is one of the most important factors for osteoporosis [4]. According to statistics, about 200 million people worldwide suffer from osteoporosis; this disease has become a serious health problem and needs to be solved urgently [5]. Studies have pointed out that estrogen can prevent bone loss and effectively relieve osteoporosis, but its long-term use may cause a series of diseases such as breast cancer and thrombosis [6]. Therefore, it is significant to find or develop drugs to treat osteoporosis.

Clinically, it’s widely reported that green tea drinking plays a positive pole in bone health [7]. For example, people with the habit of green tea drinking have a lower hip fracture rate and a higher bone mineral density (BMD) [8]. Hegarty et al. also showed that BMD at the lumbar spine is about 5% higher in tea drinkers than that in non-tea drinkers [9]. Epigallocatechin gallate (EGCG) is a natural catechin monomer obtained from green tea [10]. This component is the most bioactive in green tea, which enriches catechin, and its role in the treatment of cancer has been deeply studied [11]. In recent years, increasing studies have reported the alleviation of osteoporosis by EGCG. Lin et al. found that EGCG could accelerate BMP-2 expression in bones, thus strengthening the formation of bone matrix and ultimately promoting fracture healing [12]. Xi et al. [13] revealed that EGCG also notably induced cyclin D1, β-catenin and Wnt protein expression in mice with secondary osteoporosis, and inhibited peroxisome proliferator-activated receptor γ protein expression; in general, EGCG showed a protective effect on a mouse model of secondary osteoporosis through the Wnt/β-catenin signaling pathway. However, the relationship between EGCG and TNF-α, and the mechanism of EGCG have not been mentioned and clarified in previous studies.

LncRNAs, more than 200 nucleotides in length, are large non-coding RNAs, affecting a variety of life activities [14], including epigenetic regulation, dosage compensation, and cell differentiation regulation. Studies have reported that osteoblast differentiation can be promoted by a variety of lncRNAs, and therefore it is supposed that the lncRNAs can be used to treat osteoporosis [15]. For example, Fu et al. Showed that lncRNA ROR downregulation may inhibit osteoblast MC3T3-E1 proliferation by targeting miR-145-5p [16]. In addition, studies have pointed out that lncRNA TUG1 (TUG1) promotes vascular smooth muscle cell proliferation by regulating the miRNA-21/PTEN axis [17]. Also, Liu et al. pointed out that TUG1 promoted osteoblast proliferation and differentiation through the Wnt/β-catenin signaling pathway [18]. These studies suggest that TUG1 has the function of promoting osteoblast proliferation and differentiation. However, the specific relationship and roles of EGCG, TUG1, and TNF-α in osteoblasts are still unknown. Given this lack, this study focused on investigating the relationship by culturing mouse embryo osteoblast precursor cell line MC3T3-E1 *in vitro*, aiming to provide an effective data basis for finding drugs to prevent osteoporosis.

## 2. Material and methods

### 2.1. The culture and treatments of cells

MC3T3-E1 cells purchased from the Cell Bank of the Chinese Academy of Sciences were cultured in α-MEM complete medium in a 37°C, 5% CO_2_ incubator. To observe the roles of TNF-α, the cells were treated with 2.5 ng/mL, 5 ng/ml, and 10 ng/mL TNF-α for 72 h. For testing the therapeutic efficacy of EGCG, cells were treated with 5 μM, 10 μM or 20 μM EGCG for 1 h, and then treated with TNF-α for 72 h.

TUG1 overexpression vector pcDNA3.1-TUG1 (TUG1) and empty pcDNA3.1 (Vector), TUG1 siRNA (si-TUG1) and control siRNA (siNC) were synthesized by GenePharma (Shanghai, China). After transfection of the above vector or fragment into MC3T3-E1 cells using lipofectamine 2000 (Sigma, USA), the cells were intervened with 10 ng/ml of TNF-α for 72 h. Alternatively, cells were transfected with TUG1 siRNA after treatment with 20 μM EGCG for 1 h and cells were intervened with 10 ng/mL of TNF-α for 72 h.

### 2.2. MTT detection

A 96-well plate was applied to seed the treated cells in the logarithmic growth phase with a density of 5000 cells/well for the culture of 24 h, 48 h, 72 h. Then 20 μL of 5 mg/ml MTT solution was put into each group of cells, with culturing for another 4 h. After aspirating the supernatant, 150 μL of DMSO was put into the plate, and then 15-minute shake was conducted. A microplate reader was adopted to check the absorbance at a wavelength of 570 nm in each well.

### 2.3. Neutral red uptake assay

After 72 h of TNF-α intervention in the cells, the cell supernatants of the 0 ng/mL, 2.5 ng/ml, 5 ng/ml, and 10 ng/ml groups were aspirated. After washing cells with PBS, the neutral red uptake was measured according to the instructions of the neutral red cell proliferation assay kit. The absorbance was determined through a microplate reader (λ = 540 nm), and the neutral red uptake rate was calculated to indirectly reflect the integrity of the cell membrane and the relative cell proliferation rate.

### 2.4. Flow cytometry detection

On completion of digestion using trypsin, the cells in each group were placed into a centrifuge tube and then rinsed with pre-cooled sterile PBS twice. Subsequently, 200 μL cell suspension with concentration adjusted to 5 × 10^5^ cells/mL was put with 10 μL Annexin V-FITC and 10 μL of 20 mg/L PI solution, followed by 10 min incubation protected from light at room temperature. After that, 500 μL PBS was added. Flow cytometry was applied to check apoptosis.

### 2.5. Alkaline phosphatase (ALP) staining

The supernatant of the treated cells in each group was aspirated, followed by rinsing step of the cells. The original medium was replaced by an osteoblastic induction medium (α-MEM medium added with 100 nm dexamethasone, 10 mm β-glycerol phosphate and 50 μM ascorbic acid). The medium was altered every 3 days and ALP staining was conducted on the 7^th^ day. Afterward, the supernatant was aspirated, followed by rinsing step of the cells using PBS. Next, 30 min fixation using 4% paraformaldehyde followed PBS rinsing again. After that, the fixed cells were added with an appropriate amount of BCIP/NBT staining solution, which was prepared according to the instructions of the kit and incubated in the dark at ambient temperature for 25 min. The BCIP/NBT staining solution was then removed, and the reaction was terminated by washing with distilled water twice. A microscope was utilized for the observation of the staining, and the activity of ALP in cells was quantitatively analyzed.

### 2.6. Alizarin red staining

Osteoblastic differentiation of cells was induced for 21 days. When the cells were differentiated and matured, they were washed 3 times with 37°C PBS solution after the culture medium was removed. Next, 15 min fixation using 4% paraformaldehyde followed PBS rinsing for 3 times. After that, the fixed cells were added with an appropriate amount of 2% alizarin red staining solution for 15-minute incubation at ambient temperature for 15 min. The staining solution was removed, and the cells were rinsed with PBS 3 times. Finally, the formation of mineralized nodules was observed under a microscope.

### 2.7. qRT-PCR

Total RNA in the cells was obtained by the TRizol method, and then NanoDro was applied to determine the purity and concentration of the RNA. The random primer reverse transcription kit (Thermo, USA) was adopted for the preparation of cDNA. The level of lncRNA and mRNA expression was determined based on the instructions of the SYBR GREEN kit (TaKaRa, Japan). GAPDH was utilized as the internal reference control, and the trial was set up with 6 replicates. The calculation of the relative expression of the target gene was conducted with 2^−ΔΔCt^ method. The primer sequences were displayed in Table 1.

**Table 1.** Primer sequences

RNA	Sequences(5’ to 3’)
lncRNA TUG1	F: 5’- CTATACTCAGCTTCAGTGTT
	R: 5’- TACTGTATGGCCACCACTCC
Runx2	F: 5’- CCTGAACTCTGCACCAAGTCCT
	R: 5’- TCATCTGGCTCAGATAGGAGGG
OPN	F: 5’- AGCTCGTCTTCACCGTCAAGGA
	R: 5’- CCAGCAGATCAGGAAAGCGATG
OCN	F: 5’- CGCTACCTGTATCAATGGCTGG
	R: 5’- CTCCTGAAAGCCGATGTGGTCA
BMP2	F: 5’- TGTATCGCAGGCACTCAGGTCA
	R: 5’- CCACTCGTTTCTGGTAGTTCTTC
GAPDH	F: 5’- CTGGGCTACACTGAGCACC
	R: 5’- AGTGGTCGTTGAGGGCAATG

### 2.8. Western blot

The total proteins were extracted by RIPA (Beyotime, China). And the supernatant was also transferred to a new 200 μL centrifuge tube. The concentration of the extracted protein was measured with the BCA kit, and 20 μg of protein was taken out and then added 1× loading buffer to boil for denaturation. After that, the protein was separated through SDS-PAGE, and transferred to the PVDF membrane. After the membrane was sealed with 5% of skimmed milk for 1 h, the primary antibodies were added for co-incubation at 4°C overnight. Then, the membrane was washed for 3 times, and secondary antibody was added for another one-hour incubation at ambient temperature. Subsequently, the membrane was cleaned for another 3 times. The protein was developed with the chemiluminescence reagent. And the image was obtained from the gel imaging system. The gray level of the protein bands was analyzed through Image J software. With GAPDH as the internal reference, the relative expression of the protein was calculated.

### 2.9. Statistical methods

One-way analysis of variance and independent sample T-test analysis was conducted with SPSS26.0. Mean ± standard deviation (SD) was utilized to express the results, and *P* < 0.05 is used as the criterion for a significant difference.

## 3. Results

### 3.1. TNF-α inhibits the proliferation of MC3T3-E1 cells and down-regulates the TUG1 expression

**Figure 1.**
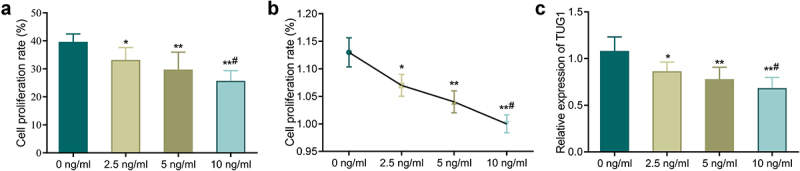
TNF-α inhibits the proliferation of MC3T3-E1 cells and down-regulates the TUG1 expression. A-B: MTT assay (a) and neutral red assay (b) detected the MC3T3-E1 cells proliferation; C: qRT-PCR measured the TUG1 expression in MC3T3-E1 cells. *P < 0.05, **P < 0.01 *vs*. 0 ng/mL group;#*P* < 0.05 *vs*. 5 ng/mL group.

First, we used different concentrations of TNF-α to treat MC3T3-E1 cells to determine the role of TNF-α. According to MTT assay and neutral red assay, the cell proliferation rate of TNF-α treated groups (2.5, 5, 10 ng/mL) was significantly reduced compared with the 0 ng/mL group (Figure 1(a,b), *P* < 0.05). Additionally, the higher the TNF-α concentration, the lower the cell proliferation rate. QRT-PCR further confirmed that TNF-α treatment could significantly decrease the TUG1 expression in a concentration-dependent manner (Figure 1(c), *p* < 0.05).

### 3.2. EGCG inhibits the effects of TNF-α on MC3T3-E1 cell proliferation and apoptosis

**Figure 2.**
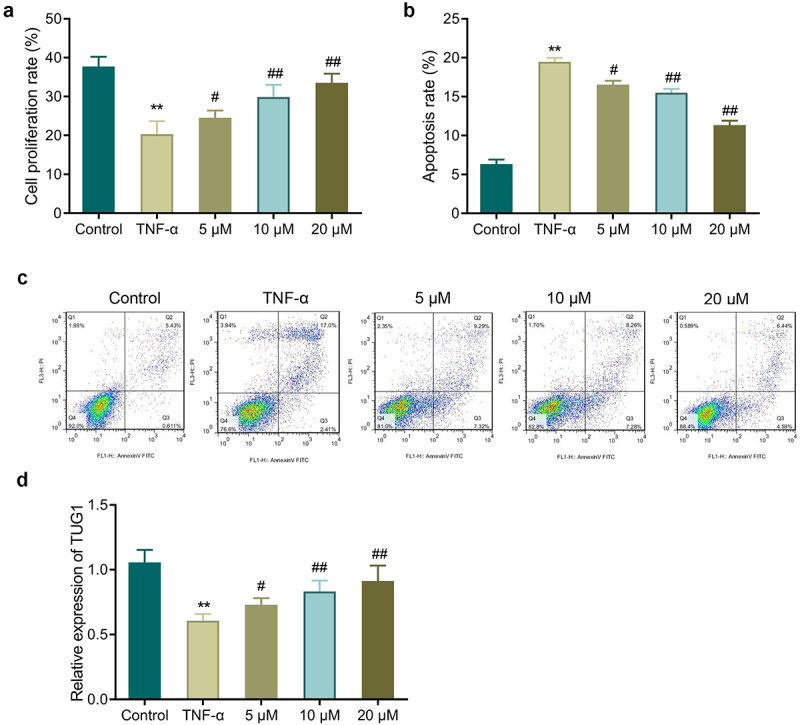
EGCG inhibits the effects of TNF-α on MC3T3-E1 cells. a: MTT assay detected MC3T3-E1 cell proliferation. b-c: Flow cytometry measured MC3T3-E1 cell apoptosis. d: TUG1 expression was determined by qRT-PCR. **P < 0.01 *vs*. Control group; #*P* < 0.05, ##*P* < 0.01 *vs*. TNF-α group.

Further, we determined the effect of EGCG on TNF-α-treated MC3T3-E1 cells. In comparison with the control group, TNF-α treated cells had a significant reduced proliferation rate while showing a marked increase in apoptosis. However, EGCG treatment could significantly increase the proliferation rate and inhibit cell apoptosis, and the higher the EGCG concentration, the more significant the improvement effect was (Figure 2(a-c), *P* < 0.05). Additionally, EGCG treatment could improve TNF-α-caused decrease of TUG1 expression, and the improvement was concentration-dependent (Figure 2(d), *p* < 0.05).

### 3.3. EGCG improves TNF-α-caused inhibition of the osteoblastic differentiation of MC3T3-E1 cells

**Figure 3.**
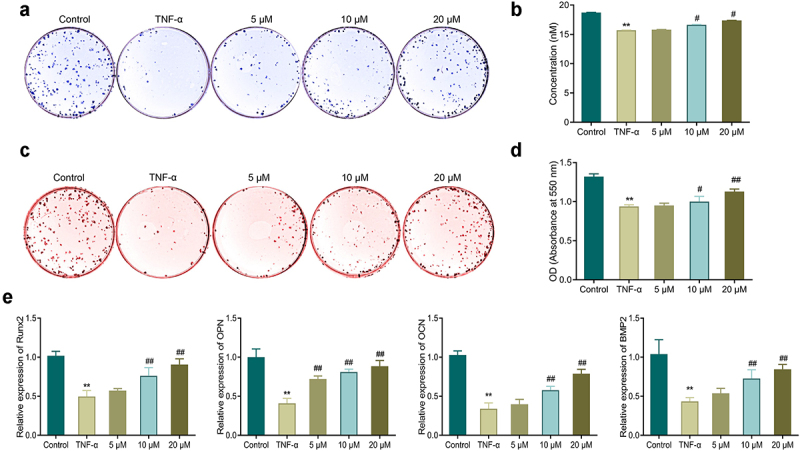
EGCG improves TNF-α-caused inhibition of osteoblastic differentiation of MC3T3-E1 cells. a-b: ALP staining for observing ALP activity in MC3T3-E1 cells. c-d: Alizarin red staining for detecting the mineralization ability of MC3T3-E1 cells. e: qRT-PCR for measuring the mRNA expression of OPN, Runx2, OCN, and Bmp2 in MC3T3-E1 cells. **P < 0.01 *vs*. Control group; #*P* < 0.05, ##*P* < 0.01 *vs*. TNF-α group.

In comparison with the control group, TNF-α treatment led to a great depression of ALP activity and mineralization ability in MC3T3-E1 cells, while a reduction in the level of osteogenic gene (Runx2, OPN, OCN, and BMP2) expression in the cells (Figure 3(a-e)). Further, the ALP activity and the mineralization ability in the cells of the EGCG treated groups were notably up-regulated in comparison with those in the TNF-α group. EGCG treatment was also able to notably up-regulate the gene level of OCN, Runx2, OPN and BMP2 in MC3T3-E1 cells. The higher the EGCG concentration was, the stronger the osteoblastic differentiation ability of MC3T3-E1 cells was.

### 3.4. TUG1 overexpression improves the effects of TNF-α on the proliferation and osteoblastic differentiation of MC3T3-E1 cells

**Figure 4.**
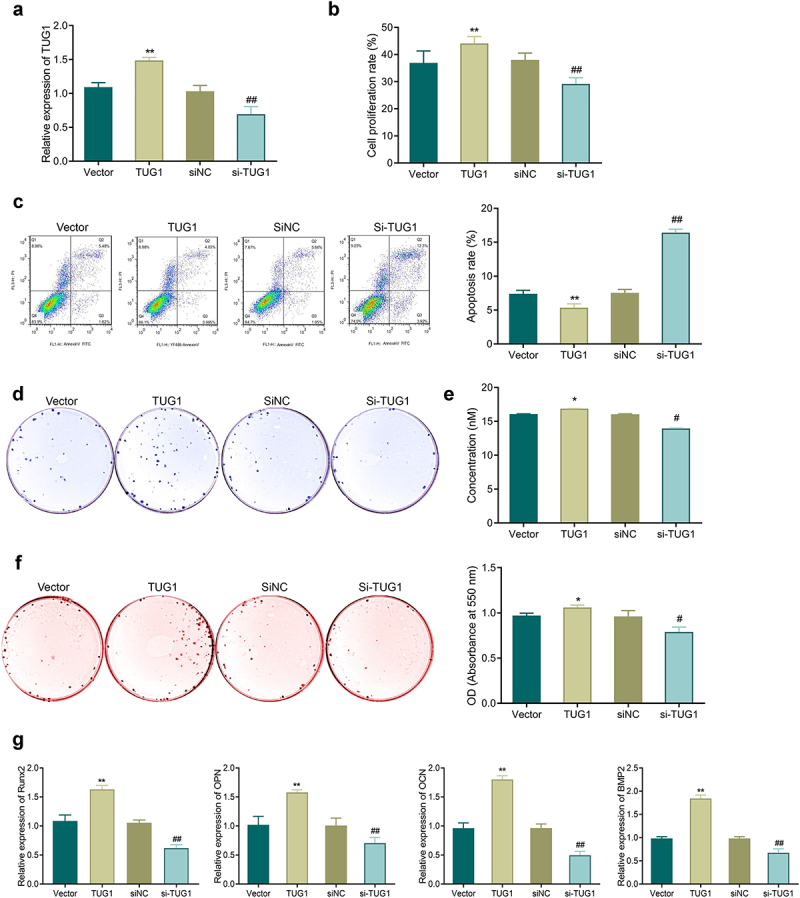
Effects of TUG1 on TNF-α-induced apoptosis, proliferation, and osteoblastic differentiation of MC3T3-E1 cells. a: qRT-PCR for detecting the efficiency of TUG1 overexpression and silencing in MC3T3-E1 cells. b: MTT assay-based measurement of the proliferation of MC3T3-E1 cells; c: Flow cytometry for detecting the apoptosis of MC3T3-E1 cells. d-e: ALP staining for observing ALP activity of MC3T3-E1 cells. f: Alizarin red staining for observing the mineralization ability of MC3T3-E1 cells, and quantitative analysis of the number of calcium nodules. g: qRT-PCR-based measurement of the mRNA expression of Runx2, OPN, OCN, and Bmp2 in MC3T3-E1 cells. *P < 0.05, **P < 0.01 *vs*. Vector group; #*P* < 0.05, ##*P* < 0.01 *vs*. siNC group.

TUG1 showed significantly higher expression in the TUG1 group while had markedly reduced expression in the si-TUG1 group (Figure 4(a), *p* < 0.05). Additionally, the TUG1 group had a higher proliferation rate and a lower apoptosis rate compared with the NC group, while the si-TUG1 group had a reduction in proliferation and an increase in apoptosis compared with the si-TUG1 group (Figure 4(b,c), *P* < 0.05). Also, transfection of pcDNA3.1-TUG1 led to a significant promotion of ALP activity and mineralization ability and an increase in the number of calcium nodules and expression of osteogenic genes; si-TUG1 transfection resulted in the opposite results as described in the TUG1 group (Figure 4(d-g)).

### 3.5. Knockdown of TUG1 diminishes the protective effect of EGCG on MC3T3-E1 cells

**Figure 5.**
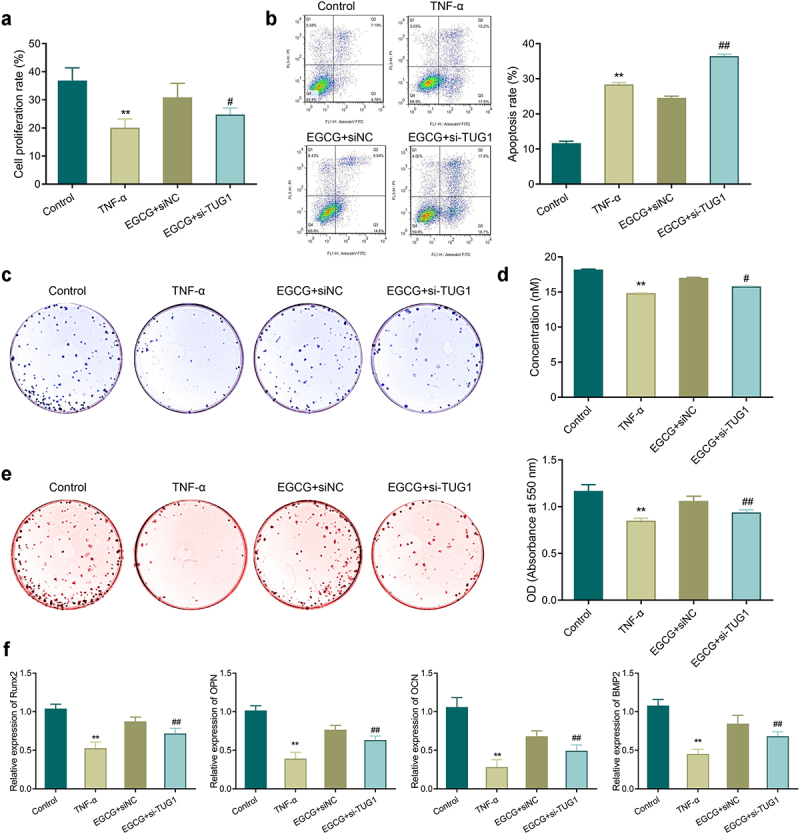
Knockdown of TUG1 can inhibit the protective effect of EGCG on MC3T3-E1 cells. A: MTT assay-based measurement of MC3T3-E1 cell proliferation; b: Flow cytometry for the detection of MC3T3-E1 cell apoptosis; c-d: ALP staining for observing ALP activity of MC3T3-E1 cells; e: Alizarin red staining for observing the mineralization ability of MC3T3-E1 cells and quantitative analysis of the number of calcium nodules. f: qRT-PCR was applied to check the mRNA expression of Runx2, OPN, OCN, and BMP2 in MC3T3-E1 cells. **P < 0.01 *vs*. Control group; #*P* < 0.05, ##*P* < 0.01 *vs*. EGCG+ siNC group.

The cell proliferation rate in the TNF-α group was notably reduced, while the apoptosis rate was increased significantly compared with the control group. Nevertheless, an increase in proliferation and a reduction in apoptosis were found after EGCG treatment, but TUG1 silencing could reverse the above effects of EGCG treatment (Figure 5(a,b)).

TNF-α treatment inhibited ALP activity and mineralization ability in the cells, and reduced the number of calcium nodules and the expression of osteogenesis-related genes significantly. EGCG treatment could enhance the osteoblastic differentiation of the TNF-α-treated cells. However, when the expression of TUG1 was silenced, the protective effect of EGCG on MC3T3-E1 cells was reversed so that inhibition of ALP activity, mineralization ability, expression of Runx2, OPN, OCN, and BMP2 could be found in the EGCG+si-TUG1 group compared with the EGCG+siNC group (Figure 5(c-f)).

### 3.6. EGCG inhibits TNF-α-caused activation of Hippo/YAP pathway in MC3T3-E1 cells

**Figure 6.**
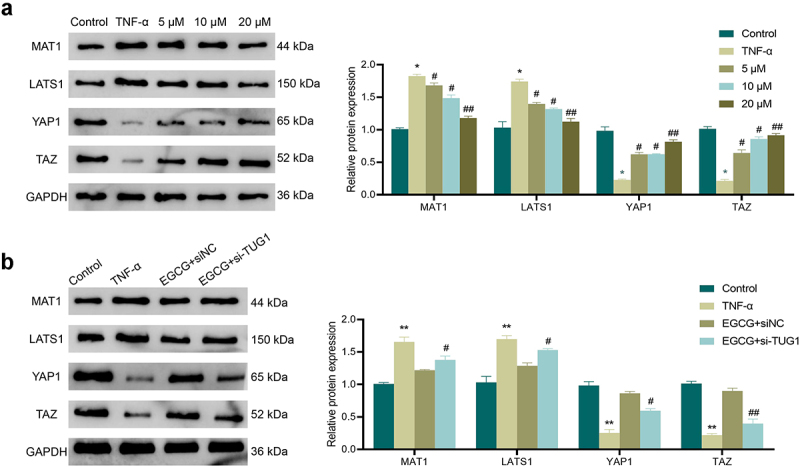
EGCG can inhibit TNF-α-caused activation of the Hippo/YAP pathway in MC3T3-E1 cells. a: Western blot was adopted to check the influence of EGCG at different concentrations of 5 μM, 10 μM, 20 μM on the expression of Hippo/YAP pathway-related proteins MAT1, LATS1, YAP1, and TAZ. *P < 0.05, **P < 0.01 *vs*. Control group; #*P* < 0.05, ##*P* < 0.01 *vs*. TNF-α group. b: Western blot was adopted for the test of the influence of different TUG1 expression and EGCG on the expression of Hippo/YAP pathway-related proteins MAT1, LATS1, YAP1, and TAZ. **P < 0.01 *vs*. Control group; #*P* < 0.05, ##*P* < 0.01 *vs*. EGCG+ siNC group.

It was shown that the Hippo/YAP signaling pathway was involved in regulating osteoblastic differentiation of MC3T3-E1 cells [19]. Therefore, we further explored the molecular mechanism of EGCG action on TNF-α-induced MC3T3-E1 cells. The results showed (Figure 6(a)) that the expression of MAT1 and LATS1 were significantly increased and the protein expression of YAP1 and TAZ were significantly lower in the TNF-α-treated cells compared with the Control group. This indicated that the Hippo/YAP signaling pathway was activated by TNF-α. In contrast, EGCG treatment significantly decreased the proteins of MAT1 and LATS1 and upregulated the proteins of YAP1 and TAZ in cells in a concentration-dependent manner. In addition, interference with TUG1 expression diminished the inhibitory effect of EGCG on TNF-α-induced Hippo/YAP signaling pathway activity in MC3T3-E1 cells (Figure 6(b)).

## 4. Discussion

Studies have pointed out that TNF-α is an osteoblastic differentiation inhibitor and osteoclastogenesis activator. Gilbert et al. stated that TNF-α inhibited osteoblast and MC3T3-E1 cell differentiation by inhibiting the expression of insulin-like growth factor I (IGF-1) [20]. Abbas et al. revealed that TNF-α suppressed osteoblast differentiation of mouse primary stromal cells through TNFR1 and Runx2 [21]. A study by Zhao et al. also showed that TNF-α transgenic mice, TNF-α caused an inhibition of the differentiation of mesenchymal stem cells (MSCs) into osteoblasts by ubiquitin E3 ligase Wwp1 [22]. Collectively, TNF-α can exert its inhibitory function on the osteoblastic differentiation ability of precursor cells via a variety of ways. In this study, we first found that TNF-α inhibited MC3T3-E1 cells proliferation and differentiation, and promoted apoptosis.

In addition, our results showed that TNF-α significantly decreased the expression of TUG1 in MC3T3-E1 cells. TUG1, as an important lncRNA that regulates cell proliferation and differentiation, has been confirmed to be able to affect the osteoblastic differentiation of a variety of cells. Existing studies have demonstrated that, TUG1, high expression in calcified aortic valve disease, can play a role of sponge to adsorb miR-204-5p, up-regulate Runx2 expression, and promote osteoblastic differentiation in CAVD [23]. TUG1 with upregulated expression in tendon stem/progenitor cells (TSPC) can also strengthen the osteoblastic differentiation of TSPC by promoting the ubiquitination of bFGF [24]. These findings suggest TUG1 can up-regulate the osteoblastic differentiation ability of cells. In the current study, up-regulating TUG1 reversed the effects of TNF-α on the apoptosis, proliferation, and osteoblastic differentiation of MC3T3-E1 cells. So it can be speculated that TNF-α-caused decrease of MC3T3-E1 cell proliferation is related to the down-regulation of TUG1 expression.

EGCG is a highly effective class of antioxidants with potential medical value, so it has received numerous attention for a long time. Past animal research has discovered that green tea polyphenol extracts has positive effects on aging process, estrogen deficiency and chronic inflammation [10]. A large number of clinical and animal researchers have also shown that bone mass can be increased and osteoporotic fractures can be reduced through tea intake, but it still keeps unknown on the relationship between tea intake and fracture healing [25]. In this study, we found that EGCG could inhibit TNF-α-caused proliferation rate decrease and apoptosis rate increase of MC3T3-E1 cells, and improve TNF-α-induced inhibition in osteoblastic differentiation. Additionally, EGCG could up-regulated TUG1 expression in the TNF-α-induced cells. And TUG1 silencing would weaken the effect of EGCG. In other research results, EGCG regulates different lncRNAs; EGCG treatment leads to changes in 15 gene expression and 285 lncRNAs associated with cholesterol metabolism in HepG2 cells [26]. And the treatment of EGCG in non-small cell lung cancer (NSCLC) cells stimulates the sensitivity of NSCLC cells to chemotherapy drugs by increasing lncRNA NEAT1 expression [27].

Finally, we explored the molecular mechanisms underlying the effects of EGCG on TNF-α-induced MC3T3-E1 cells. Studies have pointed out that TNF-α can promote Hippo/Yap signaling pathway activation [28]. Wang et al. [29] shown that TNF-α could affect osteoblastic differentiation of bone marrow mesenchymal stem cells (MSCs) by regulating the Hippo/YAP signaling pathway. Nie et al. [30] also found that dasatinib could significantly inhibit YAP expression, thereby promoting the osteoblastic differentiation of MSCs. In the study of Zhang et al., RAMP1 stimulated the Hippo/Yap pathway to enhance the osteoblastic differentiation of bone marrow MSCs [31]. The above results indicated that Hippo/YAP pathway was one of the vital signaling pathways for osteoblastic differentiation. Our study got a consistent result. We also found that EGCG could inhibit TNF-α-caused activation of Hippo/YAP pathway in MC3T3-E1 cells. However, knockdown TUG1 could reverse the effect of EGCG. Other studies have observed that both EGCG and TUG1 have a regulatory effect on the Hippo/YAP pathway [32,33].

## 5. Conclusions

In conclusion, TNF-α can activate the activity of the Hippo/YAP pathway, leading to a decrease in the proliferation rate of MC3T3-E1 cells and osteoblastic differentiation, and an increase in apoptosis rate. EGCG up-regulates TUG1 to inhibit the Hippo/YAP pathway activity caused by TNF-α and to improve biological functions of MC3T3-E1 cells, suggesting EGCG can effectively alleviate TNF-α-caused adverse effects in osteoporosis. However, the role and mechanism of EGCG have not been fully explored, and the detailed relationship between EGCG and TUG1 is currently unclear. Further investigation is still required to provide a more accurate basis for the clinical use of EGCG.
